# ALCAPA: repaired, yet at persisting risk—rediscovering a patient with a late complication

**DOI:** 10.1093/ehjimp/qyag006

**Published:** 2026-01-12

**Authors:** W Saleh, E A F Mahtab, H W Vliegen, A D Egorova

**Affiliations:** Center for Congenital Heart Disease Amsterdam-Leiden (CAHAL), Leiden University Medical Center, Albinusdreef 2, Leiden, 2333 ZA, The Netherlands; Department of Cardiology, Leiden University Medical Center, Albinusdreef 2, Leiden, 2333 ZA, The Netherlands; Department of Cardiothoracic Surgery, Leiden University Medical Center, Albinusdreef 2, Leiden, 2333 ZA, The Netherlands; Center for Congenital Heart Disease Amsterdam-Leiden (CAHAL), Leiden University Medical Center, Albinusdreef 2, Leiden, 2333 ZA, The Netherlands; Department of Cardiology, Leiden University Medical Center, Albinusdreef 2, Leiden, 2333 ZA, The Netherlands; Center for Congenital Heart Disease Amsterdam-Leiden (CAHAL), Leiden University Medical Center, Albinusdreef 2, Leiden, 2333 ZA, The Netherlands; Department of Cardiology, Leiden University Medical Center, Albinusdreef 2, Leiden, 2333 ZA, The Netherlands

A 42-year-old patient with a history of surgical correction of an anomalous left coronary artery arising from the pulmonary artery (ALCAPA) at 12 years of age was seen in the outpatient clinic after decades of loss to follow-up. She was asymptomatic and her resting electrocardiogram and transthoracic echocardiogram were unremarkable. Computed tomography angiography revealed a sharply angulated origin of the re-implanted left main coronary artery (LM), with a proximal interarterial course and critical stenosis of the pinched origin of the LM (Panels *A* and *B*). Adenosine-stress rubidium-82 positron emission tomography (PET) revealed extensive reversible ischaemia in the anterior, septal and lateral segments, corresponding to the effluence territories of the left anterior descending and circumflex arteries (LAD, LCX, Panel *C*). Coronary angiography confirmed a sub-total, long LM stenosis with flow through extensive collaterals (Panel *D*, asterisk) and a small LCX running in the atrioventricular sulcus with a small marginal artery to the lateral wall. Ancillary intracoronary imaging was not performed due to the critical obstruction (see Supplementary data online, *Video S1*). The patient underwent an extracorporeal circulation-assisted beating-heart coronary artery bypass grafting of the left internal mammary artery—LAD. The non-dominant LCX was perfused through the collaterals from the LAD and the right coronary artery, pre-operative echocardiography showed reduced longitudinal strain in the segments supplied by the LAD, with normalization of strain post-operatively. The post-operative course was unremarkable and she was discharged on the fifth post-operative day. Patient will undergo pro-active follow-up with a stress PET scheduled at 1 year post-operatively. This incidental finding of a critical LM stenosis late after ALCAPA re-implantation highlights the importance of long-term post-operative surveillance, despite the absence of symptoms.

**Figure:**
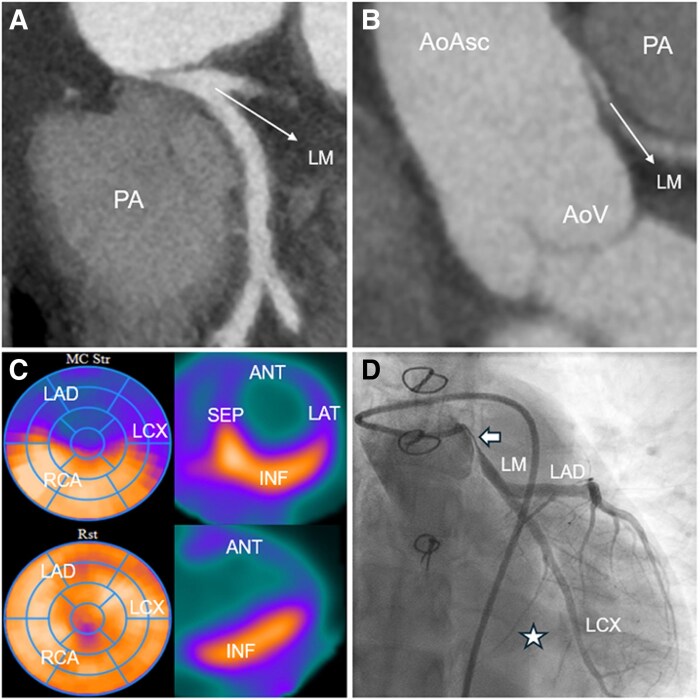


## Supplementary Material

qyag006_Supplementary_Data

## Data Availability

No new data were generated or analysed in support of this research.

## Lead author biography

Walid Saleh, MD, is a cardiology resident at Medisch Spectrum Twente (MST) and a PhD candidate at the Leiden University Medical Centre (LUMC), part of the centre for Congenital Heart Disease Amsterdam-Leiden (CAHAL). The focus of his PhD is gaining a better understanding into the hemodynamics and clinical course of patients with coronary anomalies and those with an *Anomalous Aortic Origin of a Coronary Artery (AAOCA)*, specifically.

